# Alternative Splice in Alternative Lice

**DOI:** 10.1093/molbev/msv151

**Published:** 2015-07-13

**Authors:** Jaime M. Tovar-Corona, Atahualpa Castillo-Morales, Lu Chen, Brett P. Olds, John M. Clark, Stuart E. Reynolds, Barry R. Pittendrigh, Edward J. Feil, Araxi O. Urrutia

**Affiliations:** ^1^Department of Biology and Biochemistry, University of Bath, Bath, United Kingdom; ^2^Milner Centre, University of Bath, Bath, UK; ^3^Human Genetics, Wellcome Trust Sanger Institute, Genome Campus, Hinxton, United Kingdom; ^4^Department of Animal Biology, University of Illinois at Urbana-Champaign; ^5^Department of Biological Sciences, University of Notre Dame; ^6^Department of Veterinary & Animal Science, University of Massachusetts, Amherst; ^7^Department of Entomology, University of Illinois at Urbana-Champaign

**Keywords:** head lice, body lice, alternative splicing, phenotype evolution, human parasite, transcriptomics

## Abstract

Genomic and transcriptomics analyses have revealed human head and body lice to be almost genetically identical; although con-specific, they nevertheless occupy distinct ecological niches and have differing feeding patterns. Most importantly, while head lice are not known to be vector competent, body lice can transmit three serious bacterial diseases; epidemictyphus, trench fever, and relapsing fever. In order to gain insights into the molecular bases for these differences, we analyzed alternative splicing (AS) using next-generation sequencing data for one strain of head lice and one strain of body lice. We identified a total of 3,598 AS events which were head or body lice specific. Exon skipping AS events were overrepresented among both head and body lice, whereas intron retention events were underrepresented in both. However, both the enrichment of exon skipping and the underrepresentation of intron retention are significantly stronger in body lice compared with head lice. Genes containing body louse-specific AS events were found to be significantly enriched for functions associated with development of the nervous system, salivary gland, trachea, and ovarian follicle cells, as well as regulation of transcription. In contrast, no functional categories were overrepresented among genes with head louse-specific AS events. Together, our results constitute the first evidence for transcript pool differences in head and body lice, providing insights into molecular adaptations that enabled human lice to adapt to clothing, and representing a powerful illustration of the pivotal role AS can play in functional adaptation.

## Introduction

Sucking lice are obligate hematophagous ectoparasites of placental mammals with direct life cycles, meaning they infect only a single host species (Durden and Musser 1994). This high degree of specialism and host dependence leads to long-term coevolution, such that speciation events of the host and parasite are congruent (Paterson and Gray 1997). Humans represent a special case as they are parasitized by three different types of lice, each of them colonizing a specific region of the body (head, body, and pubic area) (Reed et al. 2007; Weiss 2009). Two of these types are now usually considered members of a single species, *Pediculus humanus* which has been subclassified into *P. humanus capitis* (known as head louse) and *P. humanus humanus* (also known as *P. humanus corporis* or body louse) (Light et al. 2008). The association between the louse *P. humanus* and its human host is an ancient one, extending back at least 6 My to the last common ancestor of humans and chimpanzees. Although head lice correspond to the ancestral lineage, body lice appear to have emerged from within the head louse clade relatively recently, and probably on multiple occasions (Li et al. 2010). As the female body louse lays eggs exclusively on the host’s clothing (Light et al. 2008) it is thought that body lice first emerged after the use of clothing became widespread, approximately 170,000 years ago (Kittler et al. 2003; Toups et al. 2011). In contrast, the pubic louse, *Pthirus pubis* (not considered further in this article) is thought to have descended from the *Pthirus gorillae* which parasitizes gorillas (Reed et al. 2007), colonizing humans at some point after they lost most of their corporeal hair and *P. humanus* became restricted to the head area.

Head and body lice differ markedly in their ecological traits and were originally classified as distinct species in the mid-18th century (De Geer 1767). However, this distinction is highly contentious (Nuttall 1940; Buxton 1947). While head and body lice are not known to interbreed in the wild (Schaefer 1978; Busvine 2009), they can produce fertile offspring under experimental conditions (Mullen and Durden 2002; Bacot 2009). Furthermore, although microsatellite data have been used to argue that head and body lice coinfecting a single human host do indeed represent distinct species (Leo et al. 2005), most of the molecular data available do not support this view. For example, a comparison of ten *Cytochrome oxidase I* (*COI*) gene haplotypes representing both groups pointed to a single species (Leo et al. 2002), and this conclusion was supported by subsequent analysis of six gene loci (Light et al. 2008). Phylogenetic analysis of global samples of head and body lice, based on *18s rRNA* (Leo and Barker 2005), *COI* and *Cytochrome b* (Reed et al. 2004), and intergenic spacers (Li et al. 2010; Veracx et al. 2012), all support the view that the two groups are not phylogenetically distinct or monophyletic, and provide evidence for ongoing gene-flow between them. Thus, despite the fact that body lice have distinct morphological and ecological characters compared with head lice, there remains little justification for considering head and body lice separate species as opposed to different ecotypes of a single species.

Regardless of the taxonomic debates, the different niches occupied by head and body lice have significant relevance to public health. Head lice are very common in contemporary populations, particularly in children. Body lice have also been very common historically, but in modern times are more rarely encountered and tend to be restricted to adults living in poor sanitary conditions, such as the homeless or warring soldiers. Additionally, while head lice are not known to be vectors for infectious agents, body lice are competent vectors of three serious human pathogens; *Rickettsia prowazekii* (the causative agent of epidemic typhus), *Bartonella quintana* (trench fever), and *Borrelia recurrentis* (relapsing fever) (Rydkina et al. 1999; Weiss 2009).

The recent sequencing of the body louse genome, along with its primary endosymbiont, represents a significant advance in understanding both the ecology and evolution of this ectoparasite in relation to other insect species (Kirkness et al. 2010; Clark et al. 2013; Pittendrigh et al. 2013; Johnson et al. 2014). These efforts, however, have shed little light on the differences in ecology and vector competence observed between head and body lice. The louse genome is the smallest sequenced insect genome, being only 108 Mb; 90% of the annotated genes share homologues in other insect species (Pittendrigh et al. 2006; Johnston et al. 2007; Kirkness et al. 2010), and expressed sequence tag (EST) data have confirmed that all transcript producing genes have been annotated in the initial genome release (Olds et al. 2012). It is also clear from EST data that the transcriptomes of head and body lice are essentially identical with only one small putative difference identified to date (Olds et al. 2012; Drali et al. 2013). It has been suggested that the phenotypic shifts associated with the emergence of body lice are likely to be a consequence of a small number of point mutations or subtle regulatory changes, possibly epigenetic in origin, triggered by environmental cues (Li et al. 2010). Notably, variation in the *PHUM540560* gene, of unknown function, constitutes the only genetic marker identified to date which can distinguish head from body lice once they are removed from their habitat (Drali et al. 2013). Thus, the genomic features associated with the phenotypic differences between body and head lice remain unknown.

Alternative splicing (AS) is a common posttranscriptional process by which multiple distinct transcripts, and hence proteins, can be encoded from a single gene by differentially splicing exons. AS events can be classified depending on the region affected; exon skipping (ES) events are those where a whole exon is spliced out along with adjacent introns, 3S5S events are those where alternative starts or end of an exon are used and intron retention (IR) refers to events where an intron fails to be spliced out of the maturing transcript. In metazoans, ES events are the most prevalent type of splicing event (Kim et al. 2008) and these events tend to be more conserved between species than IR events, thus are likely to have the greater functional relevance (Ast 2004). AS is increasingly of interest in the study of functional innovation and adaptation as it provides the means for rapid functional innovations via very economical genomic changes (Nilsen and Graveley 2010; Chen et al. 2012). Novel AS regulatory elements can be generated with a few mutations, thus facilitating both short- and long-term evolutionary adaptation even in the absence of changes in the gene content through gene duplication and/or gene loss (Chen et al. 2012). A recent study in *Mus musculus* subspecies used next-generation sequencing data to show that AS can drive speciation events by generating transcript variation (Harr and Turner 2010). Moreover, many eusocial insects show distinct castes such as workers or drones, and these different phenotypes have been shown to correspond to regulatory changes mediated by AS (Lyko et al. 2010; Bonasio et al. 2012).

Here, we characterize AS patterns in human lice using RNA sequencing data from one strain of each louse (Clark et al. 2013) in an attempt to identify AS events specific to either body or head lice and thus gain insights into the potential role of AS in the ecological, phenotypic, and disease vectoring differences observed.

## Results

### AS Events in Human Lice

**Fig. 1.**
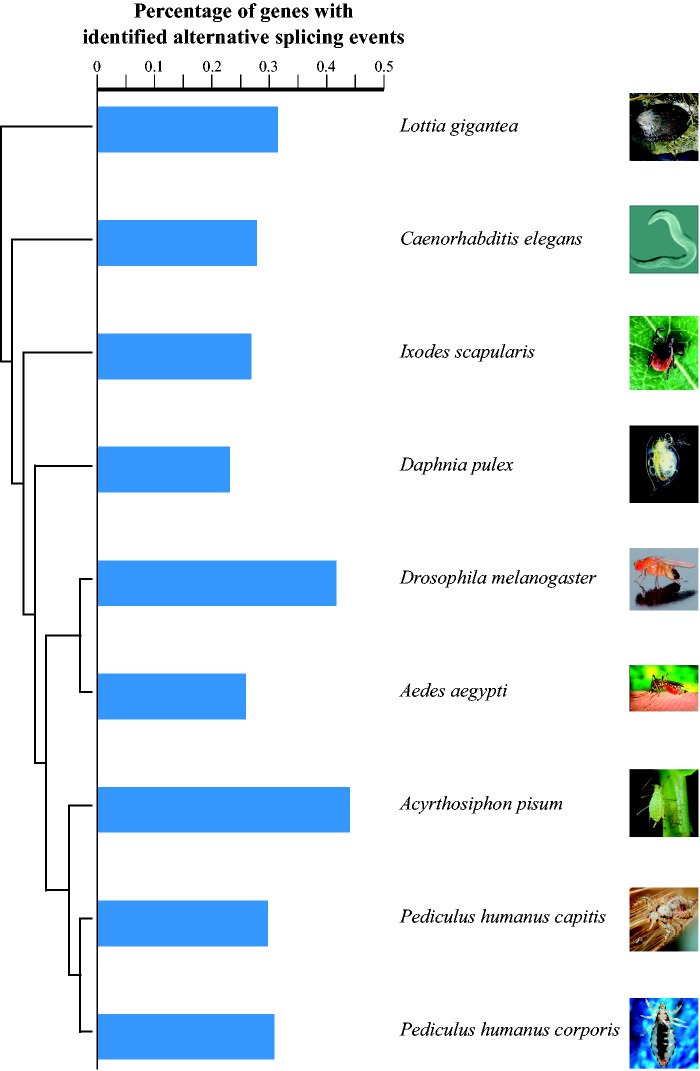
AS prevalence in human body and head lice as compared with other arthropod species. Bars adjacent to phylogenetic tree represent the proportion alternatively spliced genes in different species (the use of separate bars for head and body lice does not imply them being distinct species). *Caenorhabditis elegans* is included for reference. See supplementary table S2, Supplementary Material online, for sources of images.

In order to characterize AS patterns in human head and body lice, we analyzed *454* and short read Illumina data previously obtained from single samples of head and body lice (Olds et al. 2012). A total of 86,993 transcript sequences were found to contain one or more AS events corresponding to a total of 10,941 distinct events identified. These events can be mapped to 3,918 genes from 10,773 lice protein-coding genes. Lice have one of the smallest insect genomes known (Kirkness et al. 2010), and we investigated whether the contraction in genomic size has been accompanied by an altered overall proportion of genes undergoing AS compared with other invertebrate genomes. We obtained indexes of AS level and prevalence for lice and seven other invertebrate species transcript number normalization protocol which corrects for the bias caused by transcript coverage differences across species (see Materials and Methods). Using this comparative index we found that AS events in human lice affect about 30% of all genes, which falls within the range of AS prevalence that we observe in other arthropods (fig. 1; supplementary table S1, Supplementary Material online).

### Head and Body Louse-Specific AS Events

Of particular interest are AS events unique to either head or body lice, as such events might help to explain the molecular basis of recent adaptations of body lice to survive on clothing, or to vector bacterial pathogens. In order to explore head or body louse-specific AS events, we first used Illumina short reads obtained from the same strains of head and body lice as the *454* transcripts (Olds et al. 2012) to confirm AS events detected from *454* sequencing requiring an exact match in the genomic position of Illumina supported splice site and AS event boundaries identified from *454* transcripts. This additional data provided confirmation for 8,369 out of the total of 10,941 (76.49%) AS events identified from *454* sequences. Of these 8,369 confirmed AS events, 6,186 were observed in head louse transcripts and 6,954 in body louse. A total of 4,771 AS events were observed in both head and body lice transcriptomes with 3,598 AS events found to be unique to either head (1,415) or body lice (2,183).

**Fig. 2.**
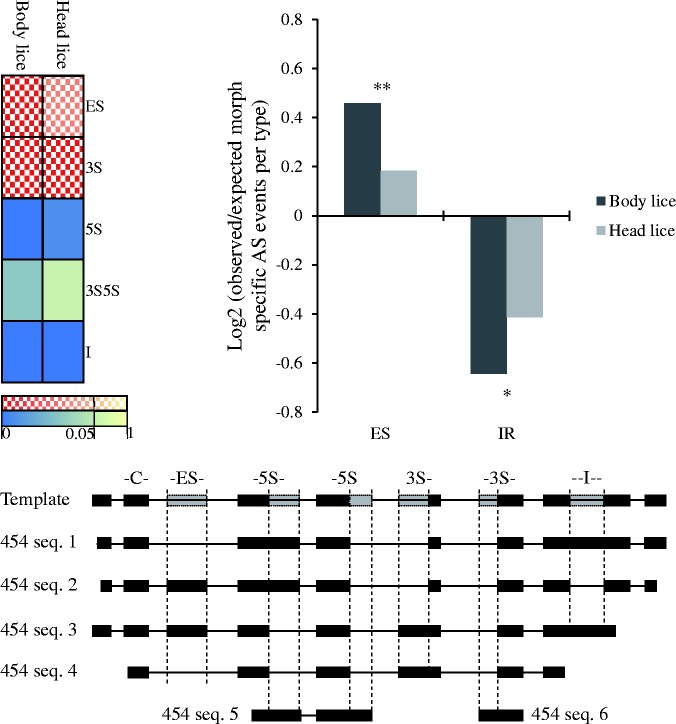
Enrichment and impoverishment of AS event types among head or body louse-specific confirmed AS events. Top left panel shows heat map graph of enrichment analysis of head and body louse-specific AS events compared with the wider pool of AS events in lice. Blocks represent enrichment (checkered blocks) and depletion (solid blocks) for each AS event type in head and body lice. Higher color intensity reflects the statistical significance of enrichment and depletion of each AS event type with yellow tones for less significant deviations from random expectation (*P* values after Bonferroni correction). All AS types deviate significantly from random expectations in both head and body lice except for 3S5S AS events. Top right panel shows significant enrichment of ES and significant depletion of IR in both head and body lice. Bottom panel shows schematic representation of AS types detected from *454* transcript sequences. AS events can be classified in five main groups ES (ES or cassette exon), 5S (alternative 5′-donor site), 3S5S (alternative 3′-acceptor and 5′-donor sites), 3S (alternative 3′-acceptor site), and I (IR). Constitutive exons are denoted by Cs.

It is possible that the higher number of body louse-specific AS events compared with the number of head louse-specific events result from increased noise in AS in body lice compared with head lice. Several studies have shown that a large proportion of AS events could be the result of splicing errors (Zhang et al. 2009; Pickrell et al. 2010). Previous studies have suggested that in metazoan genomes, ES events could be associated with a higher probability of being functionally relevant whereas IR events have been associated with lower conservation across evolution. For example, events involving the alternative use of exons (ES) are more likely to be conserved between species compared with IR events (Ast 2004). The distribution of different types of AS events common to both head and body lice, and specific to each ecotype, should therefore help us to gauge the functional relevance of these events. Using a randomization protocol (see Materials and Methods), we found that both head and body lice-specific AS events were significantly enriched in ES and alternative 3′-acceptor site (3S) AS types compared with equal-sized samples taken from the pool of all AS events identified in both or either lice (*P* = 0.0029 for ES in head lice, *P* < 0.001 for ES in body lice, and *P* < 0.001 for 3S in both head and body lice, after Bonferroni corrections; fig. 2 and supplementary tables S3 and S4, Supplementary Material online). We also found a significant underrepresentation of IR, alternative 5′-donor site (5S) and alternative 5′ donor-3′ acceptor splice sites (3S5S) for both head and body lice (*P* < 0.001 for IR and 5S in both head and body lice, *P* = 0.0770 for 3S5S in head lice, and *P* = 0.002 for 3S5S in body lice, after Bonferroni corrections; fig. 2 and supplementary tables S3 and S4, Supplementary Material online). These findings support the view that head and body louse-specific AS events contribute to the functional transcript pool, and thus are biologically meaningful.

We then compared the overrepresentation of ES and underrepresentation of IR in head and body louse-specific AS events. Body louse-specific AS events were found to have a stronger bias toward ES and depletion of IR AS events compared with head lice (2 Prop Z for ES [body vs. head] = 3.692, *P* = 0.0002, 2 Prop Z for IR [body vs. head] = −2.0192, *P* = 0.04338; fig. 2). The observation that both head and body lice have a significant bias in favor of ES type AS events while the frequency of IR AS event type is reduced in both is consistent with the view that most AS events-specific to both head and body lice are functionally relevant, however, it is worth noting that these biases are significantly more pronounced in body lice than in head lice (fig. 2).

### Enrichment of Functional Associations among Body Louse-Specific AS Events

**Fig. 3.**
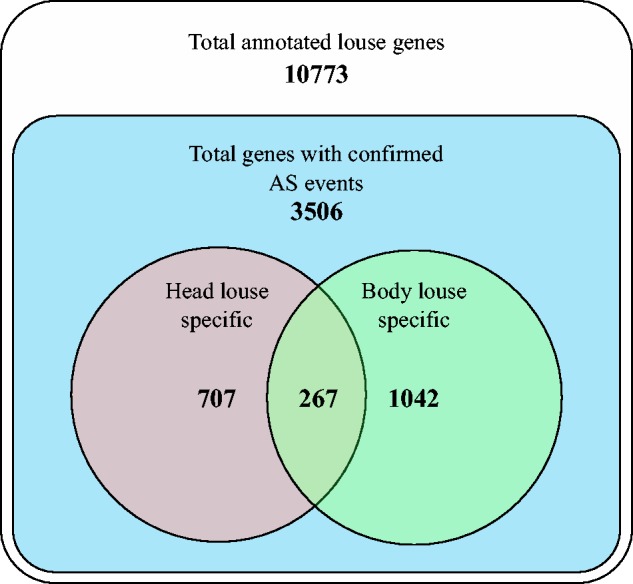
Diagram of alternatively spliced gene counts in head and body lice. The most outer square shows the total number of annotated louse genes. The inner square represents those genes with at least one confirmed AS event in either head or body lice. The left circle includes genes with at least one AS event unique to the head louse. The right set includes genes with at least one AS event unique in the body louse. The intersection includes genes with both at least one unique AS event in head lice and one unique AS event in body lice.

To assess the potential functional relevance of the inferred head or body louse-specific AS events, we examined functional associations of the sets of genes affected. The 3,598 head or body louse-specific AS events mapped to a total of 2,016 louse genes, each containing one or more AS event only found in either head or body louse derived transcripts. Of these 2,016 genes, 974 genes contained AS events unique to head lice and 1,309 contained at least one AS event specific to body lice. Of those genes containing more than one AS event, 267 were found to contain both events which were found only on head louse and also events unique to or body louse (fig. 3). As immune system genes and reproductive genes have been previously associated with processes of breeding incompatibility during speciation processes (reviewed in [Eizaguirre et al. 2009; Johnson 2010; Brucker and Bordenstein 2012]) and immune system response genes have been found to be associated with vector competence in the mosquito (Smith et al. 2011), we explored whether there was any evidence for the enrichment of genes related to immune-related function among those with head or body louse-specific AS events.

We tested for enrichment among genes with head or body louse-specific AS events using three independently compiled lists of immune-related genes (see Materials and Methods). Using a randomization test, we found no evidence of a significant enrichment of immune-related genes with either head or body louse-specific AS events when compared with the wider pool of alternatively spliced genes using all three immune gene lists tested (*P* > 0.05). We then explored the possibility that genes associated with reproductive function are overrepresented among those with AS events specific to head or body lice. For this, we used a previously compiled list of genes with reproductive function (Wasbrough et al. 2010). We found a significant enrichment of reproductive genes among those with body louse-specific AS events, but not with head louse-specific AS events (genes with head louse-specific AS *P* = 0.14; genes body louse-specific AS *P* = 0.012).

These results show that although there is no evidence that AS events disproportionately affect immunity-related genes in either head lice or body lice, there is a significant enrichment of reproductive function associated genes among those with AS events specific to the body louse.

**Fig. 4.**
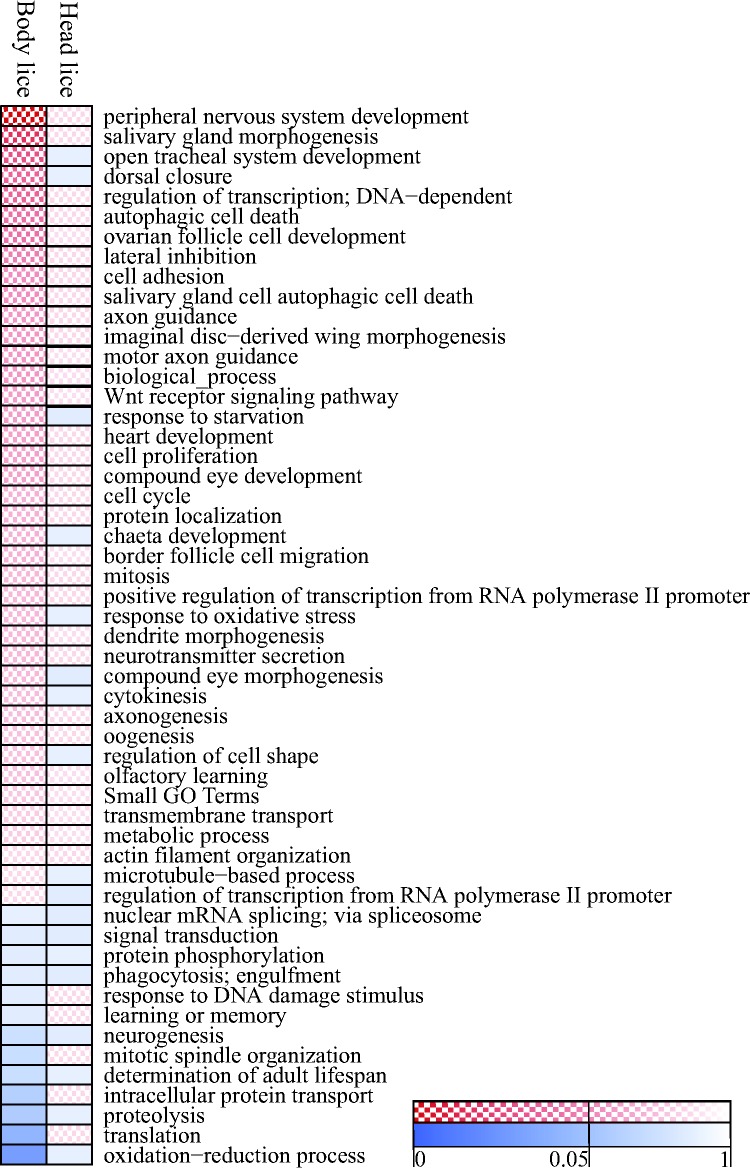
GO enrichment among genes with unique AS events in head or body lice compared with the wider set of alternatively spliced genes in either form of louse. Checkered and solid blocks represent enrichment and depletion respectively. Darker shades represent stronger statistical support for the observed variation. GO categories with fewer than 100 genes were grouped together. Side bar shows the significance threshold for overrepresentation and underrepresentation of GO categories after Benjamini–Hochberg correction.

To gain further insights into the functions of genes associated with head or body louse-specific AS events, we assigned gene ontology (GO) terms according to annotations in corresponding *Drosophila melanogaster* orthologous genes. We ascribed at least one GO term annotation for a total of 4,398 louse genes (58.7% of the total gene pool). We then examined whether any functional categories were significantly overrepresented among genes with head and body louse-specific AS events compared with the wider pool of alternatively spliced lice genes. No GO terms were found to be significantly enriched within genes with AS events unique to head lice. In contrast, genes affected by AS events unique to body lice were significantly associated with the following GO terms: “peripheral nervous system development,” “salivary gland morphogenesis,” “open tracheal system development,” “dorsal closure,” “regulation of transcription (DNA dependant),” “ovarian follicle cell development,” and “autophagic cell death” (fig. 4 and supplementary table S5, Supplementary Material online). These results show that while head louse-specific AS events affect genes representative of the wider pool of alternatively spliced genes, those in body lice show significant deviations in favor of genes associated with neuronal connectivity and development.

## Discussion

Here, we have characterized AS patterns in single human head and body lice strains by analyzing previously reported transcriptome data (Olds et al. 2012). We estimate that around 30% of louse genes are alternatively spliced, which is consistent with levels of AS found in other insects using comparable methods. We were able to identify over 3,000 AS events specific to only one type of louse.

AS provides an important mechanism for the diversification of the proteome, and changes in AS patterns have been associated with different stages of organism development (Venables et al. 2012), sex determination (Salz 2011; Venables et al. 2012), and with neural and immune system processes (Watson et al. 2005; Kurtz and Armitage 2006; Venables et al. 2012). AS has also been found to be strongly associated with transcript variation in early speciation events during a study involving mouse subspecies (Harr and Turner 2010) and with phenotypic plasticity in eusocial insects (Lyko et al. 2010; Bonasio et al. 2012; Terrapon et al. 2014), where distinct phenotypes derive from a single genome. Thus, the differences in AS events patterns in the body louse transcriptome, when compared with that of head louse, may be a consequence of adaptive evolution on the transcriptional patterns of the human louse, to colonize human clothing. An alternative explanation is that these AS events may have derived from defects in the splicing machinery, possibly resulting from the increased stress that body lice endure compared with head lice. Indeed, past studies have shown that a significant proportion of AS events identified in the human and mouse transcriptome are likely to be nonfunctional and result from splicing errors (Sorek et al. 2004; Melamud and Moult 2009; Pickrell et al. 2010; Mudge et al. 2011).

In order to test these possibilities, we characterized head and body louse-specific AS events and compared them to the wider pool of events found in both louse lineages. We first examined the distribution of head and body lice-specific AS events by type. We found a significant overrepresentation of use of alternative exon splice sites at the 3′-end (3S) and ES event types in both head and body louse-specific AS events. In contrast, the alternative 5′-end exon splice sites (5S) and IR were significantly under represented among head and body lice-specific AS events. ES events are the most common type of AS events in metazoan genomes (Keren et al. 2010) and are considered the most likely type of AS event to result in functional transcript isoforms. As many protein domains tend to be encoded within specific exons, ES events allow the production of alternatively spliced transcripts encoding proteins with distinct sets of functional domains and have thus been associated with functional genomic innovation, and a potential driver of functional complexity in metazoan lineages (Keren et al. 2010). In contrast, IR splicing events, as they result in the introduction of intronic sequence into transcripts, have a higher chance, compared with ES events, of introducing premature stop codons or frameshifts resulting in transcripts being degraded or producing nonfunctional proteins. IR events have been found to be less conserved when comparing AS across species (Ast 2004) and are generally regarded to result in fewer functional transcripts and lower impact on phenotype complexity (Kim et al. 2008). Thus, our observation that the enrichment of ES events is stronger in body lice than in head louse is consistent with stronger adaptive pressures acting on the former.

To further examine the functional relevance of AS events, we considered the functional categories of genes with AS events specific to head or body lice. If head or body louse-specific AS events are the result of neutral variation in AS patterns, or selective pressures not related to the specific challenges associated with each phenotype, then genes with head or body louse-specific AS events should be similar to random samples of the same number of genes taken from the pool of all alternatively spliced genes. Changes in the regulation of immune and reproductive associated genes may play a role in incipient speciation through the generation of mating incompatibilities (Eizaguirre et al. 2009; Johnson 2010; Brucker and Bordenstein 2012). AS in immune-related genes has also been noted to be an important factor for the interaction between parasitic microorganisms and their insect vectors (Dong et al. 2006; Smith et al. 2011). However, no significant enrichment of immune-related genes was observed among genes with AS events specific to either head or body lice related after evaluating three independently compiled lists of genes associated with immune function (Lemaitre and Hoffmann 2007; Stuart et al. 2007). There is therefore no evidence that AS can explain the increased vector competence of body lice via the modification of gene transcripts involved in immunity.

The observed enrichment of reproductive associated genes among those genes with body louse-specific AS events may be caused by several factors. First, this enrichment may result from selective pressures on reproductive-related functions, for example, adaptations of the body louse phenotype to egg-laying or changes in mating mechanisms which may vary in the human clothing environment. Alternatively, the enrichment may reflect an incipient reproductive isolation between head and body lice. Finally, the enrichment may stem from a shift in the relationship between body lice and their endosymbiotic bacteria which lice require in order to survive solely on a human blood diet (Sasaki-Fukatsu et al. 2006). The primary louse bacterial endosymbiont, *Riesia pediculicola*, is required by the host for the provision of dietary supplements, and is transmitted vertically from mother to offspring via infection of the ovarian epithelium (Sasaki-Fukatsu et al. 2006). Thus, the observed enrichment in reproductive genes could result from changes in the relationship between the louse and its endosymbiont which may contribute to adaptations relating to the adaptation of body lice to more sporadic feeding patterns in the clothing environment compared with the more continuous feeding of head lice.

Analyses of the representation of GO terms revealed that genes with body louse-specific AS events are enriched in the following GO terms: peripheral nervous system development, salivary gland morphogenesis, open tracheal system development, regulation of transcription -(DNA-dependant)-, dorsal closure, autophagic cell death, and ovarian follicle cell development. In contrast, no functional category was found to be overrepresented among genes affected by head louse-specific AS events. Interestingly, although the transcriptome data were obtained from samples of pooled individuals at different stages in the life cycles (Olds et al. 2012), all of the enriched functional categories were related to development, suggesting that some of the AS events unique to body lice may underpin some of the behavioral and morphological differences between the body and head lice strains studied.

Changes in the ovarian follicle cell development of body lice are also consistent with an enrichment in reproductive genes in body louse-specific AS events and may reflect changes in the body louse’s relationship with its endosymbiont, which must supply the larger body louse with more B vitamins (Sasaki-Fukatsu et al. 2006). Several genes with nervous system development functions have been implicated with caste differentiation in the honey bee (Barchuk et al. 2007). The significant enrichment of peripheral nervous system development functional associations among genes with body louse-specific AS events suggests a possible role for these alternative transcripts in the behavioral or sensory function differences between these two lice.

In insects and other invertebrates, autophagic programmed cell death is central to normal and stress induced development (Baehrecke 2002). In particular, this process has been shown to play an important role in early gametogenesis (Nezis et al. 2009), metamorphosis (Truman et al. 1994; Juhász et al. 2003; Rusten et al. 2004; Mirth and Riddiford 2007; Liu et al. 2009), nutrition-dependant growth rate, central nervous system structure definition (Truman et al. 1994), and remodeling of specific body structures during and between moulting (Lee and Baehrecke 2001; Juhász et al. 2003; Rusten et al. 2004). Programmed cell death-associated genes have been shown to play a role in producing alternative morphologies, including the dauer larva of *Caenorhabditis elegans,* in response to environmental stress (population density and food availability) (Liu et al. 2009), and alternative castes in the honey bee (Barchuk et al. 2007). It is thus possible that AS events specific to body lice affecting genes associated with autophagy play a role in morphological adaptations of feeding structures and reproductive organs.

Several of the enriched functions (salivary gland morphogenesis, open tracheal system development, dorsal closure, and autophagic cell death) are directly related to morphogenesis, further supporting a potential role in the development of alternative louse morphs for body louse-specific AS events. In particular, salivary gland morphogenesis and open tracheal system development might reflect feeding and or water balance adaptations in body lice phenotype compared with head lice. Notably, the salivary gland has been related to vector competence in other insects and could potentially explain the vector competence differences apparent between the two lice (Bowman et al. 1997; Gray 1998).

While the observed differences in AS between transcriptomes of head and body lice can potentially explain some of the phenotypic differences, our results do not imply that head and body lice are separate species. Instead, the observed differences in AS patterns between head and body lice may result from a small number changes in the methylation patterns of AS regulatory regions during early development, which are due to environmental cues, and which lead to differences in phenotype in the adult louse forms. This would allow for the head and body louse phenotypes to be encoded by a shared genome. Indeed, there are examples where AS patterns have been suggested to play an important role in single species phenotype diversity in eusocial insects. A link between methylation and resulting variations in AS patterns has been suggested to underlie phenotypic differences among castes in the ant *Camponotus floridanus* (Bonasio et al. 2012), the honey bee *Apis mellifera* (Jarosch et al. 2011), and termites (Terrapon et al. 2014).

It is possible that a proportion of AS events identified in this study as head or body lice-specific may in fact be common to both types of lice but failed to be assigned as such because of insufficient transcript coverage. However, the set of AS events we have identified as head or body louse specific are likely to be enriched in AS events which are indeed specific or found to be differentially expressed in future studies comparing larger transcript data sets for head and body lice and should result in similar patterns of AS type enrichment and overrepresented functional associations might be observed in future studies using larger transcript data sets.

It should also be noted that this study is based on the analysis of transcriptomes derived from a single sample for head lice and a single sample for body lice. Thus, we cannot rule out that the classification of some AS events here identified as head or body lice specific could result from between individual variations. Nevertheless, as each sample was derived from the pooling of multiple individuals at different developmental stages selected from more than one strain of head and body lice (Olds et al. 2012), the sequenced transcriptomes should be reflective of the average transcriptome for head and body lice. The fact that differential expression analysis yielded no significant differences for any gene in the original report (Olds et al. 2012), further shows the high degree of similarity between the analyzed head and body lice transcriptomes and makes in unlikely to be affected to a significant extent by between individual variations. Finally, if the differences observed were the result of between individual variability lice-specific AS events should be distributed between the two lice in a random manner. Our findings, instead, show significant differences in the overrepresentation of specific AS types. In addition, significant enrichment of developmental-related functional categories are exclusive to body lice-specific AS events.

In conclusion, the comparison of head and body louse transcriptomes, presented in this study, has revealed differences in AS patterns and the first insights into the differences in the transcript pool which may contribute to the lifestyle and vector competence variation observed for head and body lice. Head louse-specific AS events appear to largely resemble the background gene pool of alternatively spliced lice genes. In contrast, genes affected by AS events unique to body lice were significantly enriched for certain functions. This may reflect the strong directional selection in human lice underpinning adaptation to human clothing. The nature of the overrepresented GO terms, suggest that changes in the developmental program, particularly in relation to feeding and development of the nervous system may have played a role in increasing the flexibility during development allowing human lice to inhabit clothing. Confirmation of the significance of these observations would be possible through multiple comparisons of different strains of head and body lice. Finally, it is important to note that the observed differences in AS events between head and body lice do not contradict and are entirely consistent with past DNA-based reports supporting that head and body lice constitute a single species with the two lice representing alternative morphs.

## Materials and Methods

### Identifying AS Events

A total of 40,1578 partial transcript sequences obtained through *454* sequencing from a single sample for head and a single sample for body lice were analyzed (Olds et al. 2012). Each sample is made up of a pool of lice at different stages of development and corresponding to more than one strain (Olds et al. 2012). Individual transcripts were aligned to the body louse genome (Kirkness et al. 2010) using GMAP (Wu and Watanabe 2005). Reads which aligned to regions with no annotated genes were discarded from any further analysis. AS events were identified according to methods used previously (Chen et al. 2011). In brief, *454* transcripts to genome alignments were used to generate exon–intron gene templates. Then AS events were identified by comparing alignment coordinates of *454* sequences from head and body lice, respectively, against corresponding gene templates. Of the complete set of 401,578 transcript sequences (generated by *454* sequencing), a total of 86,993 contained one or more AS events corresponding to a total of 10,941 distinct events identified.

To produce comparable values of AS a random sampling method was used to correct for sequence coverage bias in both head and body lice (Kim et al. 2007) as implemented in Chen et al. (2014). In summary, the corrected AS index is considered as the average number of AS events found in 100 samples of 10 randomly selected *454* sequences aligned to the louse genome.

### Illumina Confirmation of Splice Sites

Illumina RNA-Seq short reads obtained from the same samples as the 454 sequences (Olds et al. 2012) were aligned to the genome and identified splice sites using GSNAP (Wu and Watanabe 2005). Coordinates of splice sites were then compared with the start and end positions of each AS events identified from *454* sequences. For an AS event to be counted as “confirmed,” an exact match of an Illumina identified splice site to at least one boundary (start or end) of the AS event was required.

### Functional Gene Associations

Associations to immune functions were tested using three separate lists of genes: immune response (Lemaitre and Hoffmann 2007); phagocytosis, from proteomics analysis of purified phagosomes (Stuart et al. 2007); and phagocytosis, from RNAi screening during bacterial phagocytosis (Stuart et al. 2007). GO terms were assigned according to gene orthology with *D. melanogaster* obtained from FlyBase (McQuilton et al. 2012). We obtained at least one biological process GO term annotation for a total of 4,398 louse genes (58.7%). GO terms with less than 100 annotated louse genes were grouped together.

### Randomization Tests

Overrepresentation of AS type among head or body louse-specific AS events was tested with a randomization test. For this the proportion of head or body louse-specific AS events (or their corresponding genes) associated with the feature of interest was calculated and then contrasted with randomly selected samples of equal size taken from the pool of all AS events identified in both or either lice groups. To test overrepresentation of functional associations with genes with mapping head or body louse-specific AS events, we calculated the frequency of functional associations in the test sets as well as in randomly selected samples of the same number of genes taken from the full set of genes with at least one AS event in both head and body lice or in either of the two. Statistical significance for observed enrichments or impoverishments of AS types or functional associations was then established using a randomization protocol obtaining 10,000 random samples of the same number of AS events or genes as the ones being tested for enrichment, drawn from the whole population of alternatively spliced louse genes. The number of AS events or genes associated with the feature being tested among genes with head or body louse-specific AS events is then compared with the mean of the number of genes pertaining to that same feature in the random samples by means of a *Z*-test. A Benjamini–Hochberg correction for multiple testing was then applied where needed.

## Supplementary Material

Supplementary tables S1–S5 are available at *Molecular Biology and Evolution* online (http://www.mbe.oxfordjournals.org/).

Supplementary Data
