# Supplementary material for: Alternative Splice in Alternative Lice
Source: Mol Biol Evol. 2015 Jul 13;32(10):2749–59. doi: 10.1093/molbev/msv151 (PMC4576711; doi:10.1093/molbev/msv151)
Supplement: Supplementary Data [file supp_32_10_2749__index.html]

Alternative Splice in Alternative Lice — Alternative Splice in Alternative Lice — Supplementary Data 

# Alternative Splice in Alternative Lice

## Supplementary Data

files

- Supplementary Data - pdf file
