# Supplementary material for: Alternative Splice in Alternative Lice
Source: Mol Biol Evol. 2015 Jul 13;32(10):2749–59. doi: 10.1093/molbev/msv151 (PMC4576711; doi:10.1093/molbev/msv151)
Supplement: Supplementary Data [file supp_msv151_Tovar_et_al_supplementary_information.pdf]

**SUPPLEMENTARY INFORMATION**

Supplementary Table 1. Alternative splicing (AS) prevalence in head and body lice as compared to other invertebrate species. Human and mouse are included for reference.

| Subgroup | Scientific name | Estimated AS prevalence using comparative index AS |
|---|---|---|
| Insects | *Pediculus humanus capitis* | 0.2974 |
| Insects | *Pediculus humanus corporis* | 0.3089 |
| Insects | *Drosophila melanogaster* | 0.4166 |
| Crustacea | *Daphnia pulex* | 0.2312 |
| Arachnida | *Ixodes scapularis* | 0.2683 |
| Roundworms | *Caenorhabditis elegans* | 0.2785 |
| Mammals | *Homo sapiens* | 0.8909 |
| Mammals | *Mus musculus* | 0.842 |

Supplementary Table 2. Image sources

| **Species name** | **Source** |
|---|---|
| *Pediculus humanus capitis* | Gilles San Martin, "Male human head louse" August 17, 2010 via Flickr, Creative Commons Attribution-Share Alike http://commons.wikimedia.org/wiki/File:Male_human_head_louse.jpg |
| *Pediculus humanus corporis* | CDC/ Frank Collins, Ph.D., "Pediculus humanus var. corporis" 2006 via Public Health Image Library, public domain http://phil.cdc.gov/phil/details.asp?pid=9206 |
| *Drosophila melanogaster* | André Karwath, "Drosophila melanogaster - side" July 16, 2005 via Wikipedia Commons, Creative Commons Attribution-Share Alike http://commons.wikimedia.org/wiki/File:Drosophila_melanogaster_-_side_(aka).jpg |
| *Daphnia pulex* | Paul Hebert, "Daphnia pulex" June 14, 2005 via Wikipedia Commons, Creative Commons Attribution http://commons.wikimedia.org/wiki/File:Daphnia_pulex.png |
| *Ixodes scapularis* | Scott Bauer, "Adult deer tick, Ixodes scapularis", public domain http://commons.wikimedia.org/wiki/File:Adult_deer_tick.jpg |
| *Caenorhabditis elegans* | Bob Goldstein, "Caenorhabditis elegans, adult hermaphrodite" 2007 via Wikipedia commons, Creative Commons Attribution-Share Alike http://commons.wikimedia.org/wiki/File:CelegansGoldsteinLabUNC.jpg |
| *Aedes aegypti* | James Gathany, "The yellow fever mosquito Aedes aegypti, taking a bloodmeal." 2005 via Public Health Image Library, public domain http://commons.wikimedia.org/wiki/File:Aedes_aegypti_bloodfeeding_CDC_Gathany.jpg |
| *Acyrthosiphon pisum* | Shipher Wu, "Pea aphids extracting sap from the stem and leaves of garden peas." February 2010 via Wikipedia commons, Creative Commons Attribution http://commons.wikimedia.org/wiki/File:Acyrthosiphon_pisum_(pea_aphid)-PLoS.jpg |

Supplementary Table 3. Enrichment and impoverishment of alternative splicing (AS) event types among genes with head louse specific AS events.

| AS event type | Head louse specific AS events per type | Expected number of head louse specific AS events from 10000 random samples | Standard Error | Z-score | One sided p value | Bonferroni adjusted p-value |
|---|---|---|---|---|---|---|
| ES | 398 | 350.3187 | 14.71017 | 3.2413 | 0.000594754 | 0.002973767 |
| 5S | 303 | 368.1552 | 14.95069 | -4.3580 | 6.56E-06 | 3.28E-05 |
| 3S | 329 | 208.6949 | 12.24355 | 9.8259 | 4.35E-23 | 2.18E-22 |
| I | 238 | 316.8503 | 14.1002 | -5.5921 | 1.12E-08 | 5.61E-08 |
| 3S5S | 147 | 170.9809 | 11.10434 | -2.1595 | 0.0154019 | 0.07700952 |

Supplementary Table 4. Enrichment and impoverishment of alternative splicing (AS) event types among genes with body louse specific AS events.

| AS event type | Body louse specific AS events per type | Expected number of body louse specific AS events from 10000 random samples | Standard Error | Z-score | One sided p value | Bonferroni adjusted p-value |
|---|---|---|---|---|---|---|
| ES | 742 | 540.3015 | 17.44294 | 11.56333 | 3.16E-31 | 1.58E-30 |
| 5S | 488 | 568.337 | 17.55804 | -4.57551 | 2.38E-06 | 1.19E-05 |
| 3S | 420 | 321.8113 | 14.09827 | 6.964593 | 1.65E-12 | 8.23E-12 |
| I | 313 | 489.227 | 16.78252 | -10.50063 | 4.29E-26 | 2.15E-25 |
| 3S5S | 220 | 263.3232 | 13.05875 | -3.317561 | 0.000454036 | 0.00227018 |

Supplementary Table 5. GO term categories enriched and impoverished among genes with body louse specific alternative splicing events. No terms were enriched or underrepresented in head lice.

| Enriched GO ID | GO Term | Genes with body lice specific AS events in GO Term | Expected no. of genes | Standard Error | Z-score | BH adjusted p-value |
|---|---|---|---|---|---|---|
| GO:0007422 | peripheral nervous system development | 30 | 17.2124 | 3.2522 | 3.9319 | 0.0022 |
| GO:0007435 | salivary gland morphogenesis | 26 | 15.2889 | 3.0898 | 3.4665 | 0.0069 |
| GO:0007424 | open tracheal system development | 40 | 27.6003 | 4.0973 | 3.0262 | 0.0164 |
| GO:0007391 | dorsal closure | 39 | 27.5647 | 4.1133 | 2.7800 | 0.0280 |
| GO:0006355 | regulation of transcription; DNA-dependent | 50 | 37.1758 | 4.6985 | 2.7293 | 0.0280 |
| GO:0030707 | ovarian follicle cell development | 25 | 16.807 | 3.2408 | 2.5280 | 0.0395 |
| GO:0048102 | autophagic cell death | 24 | 16.0587 | 3.1591 | 2.5137 | 0.0395 |
| Impoverished GOID | | | | | | |
| GO:0055114 | oxidation-reduction process | 26 | 41.73 | 5.0595 | -3.10898 | 0.0164 |
| GO:0006412 | translation | 12 | 20.8038 | 3.5644 | -2.46986 | 0.0397 |
